# Tendon modification with percutaneous Ultrasound-Guided Tenotomy using TENEX®: A histological and macroscopic analysis of a bovine cadaveric model

**DOI:** 10.1016/j.inpm.2025.100590

**Published:** 2025-05-08

**Authors:** Suwannika Palee, Chuanyong Lu, Adam Betcher, Ugur Yener, Jonathan Alerte, David Howarth, Luke Cooper, Asude Nur Hasanoglu, Alaa Abd-Elsayed, Sayed Emal Wahezi

**Affiliations:** a Department of Rehabilitation Medicine, Faculty of Medicine, Naresuan University, Phitsanulok, Thailand; b Department of Physical Medicine & Rehabilitation, Montefiore Medical Center, Bronx, NY, USA; c Department of Pathology, Montefiore Medical Center, Bronx, NY, USA; d New York City Football Club, New York, NY, USA; e Department of Physical Medicine and Rehabilitation, Metropolitan Hospital, New York Medical College, New York, NY, USA; f Department of Anesthesiology, University of Wisconsin, Madison, WI, USA

**Keywords:** Chronic, Tendinopathy, Paratenon, Nociceptors, Future, Pain

## Abstract

**Background:**

Chronic tendinopathy treatment remains elusive; however, percutaneous Ultrasound-Guided Tenotomy using TENEX® (PUTT) has demonstrated promising clinical outcomes, but the mechanism of action is not clearly defined. This study aims to describe potential mechanisms using an ex-vivo animal model. The objective of the study is to examine the histological effects of PUTT on the bovine soleus tendon across varying treatment durations.

**Methods:**

Twelve bovine soleus tendons were allocated to four cohorts to undergo PUTT for 1, 3, 5, and 7 min. Each specimen was treated on one side, the opposite side serving as a control. Macroscopic and microscopic analyses were conducted to assess tendon sheath, fascicle, and perineural disruption. Fascicle penetration was measured using ImageJ software. Statistical analyses were performed using Analysis of Variance (ANOVA), with significance levels set at p < 0.05.

**Results:**

Macroscopic and microscopic examination revealed progressive separation of the paratenon, peritendinous nerves, and fascicles, correlating with increased treatment duration. Fascicle penetration depths were 0.1 mm, 2.57 mm, 2.61 mm, and 3.93 mm at 1, 3, 5, and 7 min, respectively. ANOVA confirmed significant differences among groups (F (3, 8) = 620.898, p < 0.001), with a large effect size (η^2^ = 0.996. Tukey's Honest Significant Difference (HSD) test revealed significant differences between most groups (p < 0.001), except between the 3-min and 5-min treatments, which showed no significant difference (p = 0.969).

**Conclusion:**

PUTT induces significant structural changes in the paratenon and fascicle layer with longer treatment duration, resulting in more pronounced modifications.

## 1 Introduction

Despite the extensive range of therapeutic strategies available for tendinopathy—from conservative options like physical therapy and pharmacological treatments to more invasive methods such as steroid injections and surgery—the management of this prevalent condition continues to be a significant challenge [1]. Conservative treatments, preferred for their lower risk profiles, fail to alleviate symptoms in approximately 25 %–45 % of patients, often necessitating surgical intervention [2,3]. However, surgery is not suitable for all patients due to its potential complications and strict eligibility criteria. This underscores a critical need for safer and more effective treatment modalities.

Percutaneous Ultrasound-Guided Tenotomy using TENEX® (TENEX Health, Lake Forest, CA) (PUTT) has emerged since 2012 as a promising technique in this context. This minimally invasive, time-dependent cutting approach employs high-frequency ultrasound energy delivered through a dual chamber 18-gauge needle to specifically target tendon [[4], [5], [6]]. However, the effects of varying treatment duration on the paratenon and tendon fibrous tissue, both macroscopically and microscopically, remain unexplored.

This study investigates the structural changes induced by PUTT in ex vivo bovine tendons, analyzing both gross and histological modification. Most investigators use ultrasound localization to direct the tool onto its target. Numerous clinical studies have demonstrated that PUTT can significantly reduce pain and enhance functional outcomes, with success rates ranging from 80 % to 95 % [[7], [8], [9], [10], [11], [12], [13]]. However, there remains a paucity of detailed documentation regarding the histological effects of PUTT, particularly concerning the tendon structure and its surrounding peritendinous tissue. Previous research has shown that treatment durations as brief as 7 min can completely section the coracohumeral ligaments, validating significant structural changes induced by this procedure [6]. However, there is a notable lack of comprehensive studies examining the macroscopic and microscopic effects of PUTT on tendon structure.

Recent studies have increasingly focused on peri tendinous tissue (paratenon) as a source of chronic tendon pain due to its major role in nociceptive innervation, pathologic neural ingrowth, and tendon homeostasis [[14], [15], [16], [17], [18]]. Protracted neural ingrowth, particularly the prolonged proliferation of nociceptive nerve fibers that sprout from the paratenon into areas typically sparse in such fibers as the tendon proper, has been identified as a crucial factor in the pathophysiology of tendinopathic pain [19,20]. This underscores the potential for developing treatments aimed at disrupting neural outflow within the tendon. Previous authors have proposed that the clinical effects of PUTT for various chronic tendinopathies are due to fiber separation and consequential loosening of tendon architecture [7,12,21].

Presently, no studies have investigated the effects of varying durations of PUTT application on the microscopic tendon structure, and the potential mechanism of action remains unclear. It is hypothesized that PUTT mechanism of action involves modification of tendon and paratenon architecture. Therefore, the research aim of this study is to perform histological analysis of tendon and paratenon architectural changes following application of PUTT in an ex vivo bovine model.

## 2 Methods

### 2.1 Tissue preparation and sample grouping

A total of four bovine hindlimbs, with the intact Achilles tendon-calcaneus insertion and preserved proximal muscle-tendon junction, were collected from animals over 12 months of age at a local abattoir intended for human consumption. After a 24-h cooling period at −22 °F and gradual thawing in the refrigerator, the samples were removed from the unit and then allowed to reach room temperature over the next 12 h. The Achilles tendon samples were resected 2 cm distal to the musculotendinous junction and 2 cm proximal to the calcaneal insertion using a transverse cut with a number 10 scalpel, followed by the removal of the fascia and paratenon of the Achilles. Following the dissection, the Achilles tendon components, including the soleus and gastrocnemius tendons, were explored. Due to the variation in thickness and width of the gastrocnemius tendons, and in contrast, the soleus component having a uniform thickness and width over approximately 10 cm in length, it was decided to use specimens composed of the soleus component. Each soleus tendon specimen was typically 10 cm in length, with a thickness of 10 mm ± 2 in the anteroposterior direction and 15 mm ± 2 in the transverse direction, measured using a surgical ruler. From each specimen, three tendon samples, each 2 cm in length and as uniform as possible in width and thickness, were obtained, resulting in a total of 12 tendon specimens.

**Fig. 1:**
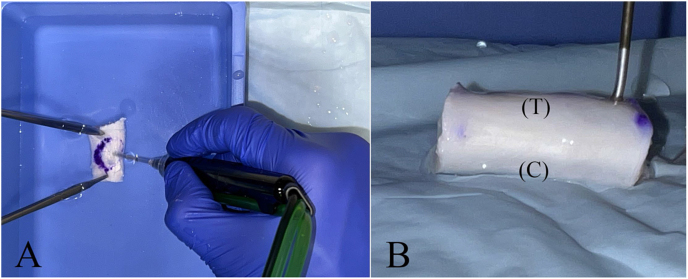
A. Application of the PUTT needle on the treated surface of the tendon. B. Markings on each tendon to indicate the treatment (T) and control (C) surfaces.

The specimens were marked on each surface to identify treatment (T) and control (C) (Fig. 1). The treated surface of each tendon underwent PUTT, while the opposite surface remained untreated to serve as a control. The specimens were placed in specimen containers immersed in 0.9 % normal saline to simulate physiologic conditions. Four experimental groups were assigned based on the time of PUTT. Three specimens from each cohort were then treated for 1, 3, 5, or 7 min on the T surface. Treatment areas were marked with a non-dissolving marker in an oval shape measuring 1 cm in diameter. PUTT was performed within this area because it matched the treatment size reported in other manuscripts The time between the completion of the sample preparation process and the start of PUTT was 10 ± 5 min.

### 2.2 Percutaneous Ultrasound Tenotomy procedure

A standard Tenex tenotomy needle was used. The needle was guided across the tendon fibers in an oscillatory motion over an area 1 cm in diameter, as described in previous clinical studies [7,12,21,22]. Two forceps stabilized the specimen during the needling process to limit the aberrant motion of the tissue.

### 2.3 Macroscopic examination

Macroscopic examinations were conducted to assess peri tendinous tissue loosening, color, and consistency changes corresponding to the duration of PUTT application. Each specimen was visually and manually inspected for texture and elasticity changes. The extent of peritendinous tissue loosening and separation was observed from the point of separation from the tendon's fibrous structure to the furthest extent of visible loosening. Pre- and post-treatment conditions of each specimen were documented with photographs taken using an iPhone 13 Pro camera (12 MP sensor, 1.9 μm pixels, 26 mm equivalent f/1.5-aperture lens).

The macroscopic analysis was performed collaboratively by four authors (SP, UY, AB, JA), who are clinicians and researchers with experience in musculoskeletal experimental design and laboratory-based studies. During the experiment, all four authors directly observed the macroscopic characteristics of each specimen and recorded their observations in real time. To ensure consistency and reduce observational bias, one experienced specialist (SW), who has prior experience in bovine Achilles tendon research, facilitated consensus discussion immediately following treatment for each specimen.

### 2.4 Microscopic analysis

Following tenotomy, marking dye was applied to the top side of each treated tissue, identifying the areas treated by PUTT to guide histologic analysis. Tissues were fixed in 10 % neutral buffered formalin for 15 days, then processed through graded alcohols and xylene using an automated Leica Tissue Processor. Paraffin embedding was performed manually in both longitudinal and cross-sectional orientations. Sections were cut from the wax blocks at 2–10 μm to yield single-cell layer sections and stained with Hematoxylin and Eosin. Microscopic analysis was performed by a Board-Certified Pathologist using the Olympus microscope system equipped with 10 mm. in 100 divisions scale bar (0.1 mm per division). This evaluation assessed characteristic changes at the treatment sites, focused on fibrous tissue and paratenon, comparing various treatment durations in both longitudinal and cross-sectional orientations of the tendon. Quantitative assessment of tendon fibrous penetration depth was performed using a protocol developed specifically for this study. A single representative histological cross-section was selected from the center of the treated region, defined by a 1 cm diameter area. Each section was processed, photographed, and analyzed using ImageJ software (version 1.54g, NIH, USA). Three linear measurements were taken from the treated tendon surface to the deepest point of visible fiber modification, and the mean of these values was recorded as the penetration depth for each specimen. Measurements were independently performed by a researcher (SP) and a pathologist (CL); any discrepancies were resolved through consensus to ensure accuracy and consistency across all treatment groups.

### 2.5 Statistical analysis

Descriptive statistics, including mean and standard deviation, were calculated for tendon fibrous tissue penetration depth. An Analysis of Variance (ANOVA) was used to assess the significance of differences across treatment durations. Significant findings were further analyzed with Tukey's HSD test for post hoc comparisons. All analyses were conducted using SPSS software, version 25 (IBM Corp., Armonk, NY), with a significance level set at p < 0.05.

## 3 Results

This investigation assessed the effects of PUTT on bovine Soleus tendons across four treatment durations: 1, 3, 5, and 7 min, analyzing three samples per treatment group. The study comprehensively evaluated both macroscopic and microscopic changes within the paratendinous tissue and collagen structure of the tendon. Each variable was thoroughly examined and documented to provide a detailed understanding of the changes induced by varying durations of PUTT application.

### 3.1 Macroscopic examination

**Fig. 2:**
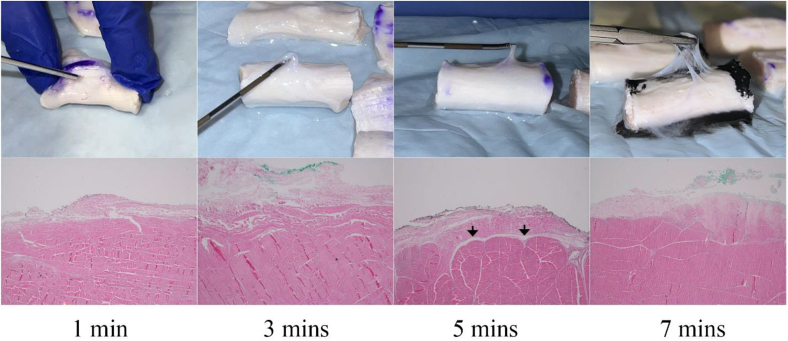
Progressive Levels of Paratenon Separation — This figure displays progressive levels of paratenon separation, providing detailed microscopic views that align with macroscopic observations. These images illustrate a time-dependent increase in the extent of separation, visually capturing the loosening of the peritendinous tissue and correlating each stage with the duration of PUTT. Arrows indicate areas of fiber misalignment and altered spacing between the peritendinous tissue and tendon fibrous structure.

During the macroscopic examination, significant changes in the peritendinous tissue and tendon consistency were observed, which varied with the duration of the TENEX® application. After 1 min of treatment, slight loosening of the peritendinous tissue was noted, with minimal changes in the tendon's original firmness and no marked separation from the tendon's fibrous structure. By 3 min, moderate loosening and the beginning of separation were visible, accompanied by a slight reduction in firmness and slight discoloration compared to the untreated side. The changes became more pronounced at 5 min, with marked loosening and clear separation, along with a noticeable decrease in tendon firmness (Fig. 2). At 7 min, the most extensive loosening occurred, with the peritendinous tissue easily distinguishable from the tendon fibrous and the tendon showing major alterations in texture and significantly less firmness. A summary of the macroscopic changes of each variable is shown in Table 1.

**Table 1:** Macroscopic changes in peritendinous tissue and tendon consistency across treatment durations.

Treatment Duration	Peritendinous tissue loosening	Peritendinous tissue separation	Discoloration	Tendon Consistency
1 min	Slight	Not evident	None	Firm, minimal change from control
3 min	Moderate	Beginning	None	Slightly less firm, noticeable softening
5 min	Marked	Clear	None	Less firm, noticeable decrease in firmness
7 min	Extensive	Easily distinguishable	None	Significantly less firm, alterations in texture

### 3.2 Microscopic analysis

**Fig. 3:**
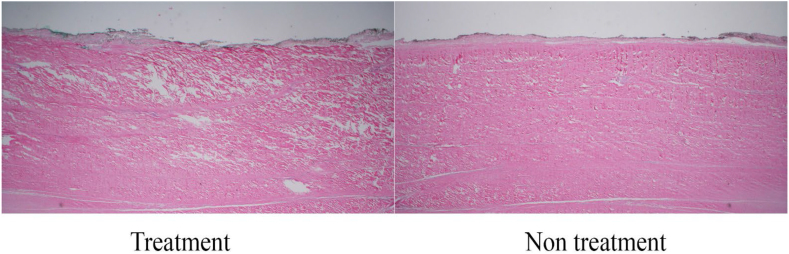
Comparison of Tendon Fibrous Tissue Modification — This image shows the structural differences in a tendon, highlighting tracts of modified fibrous tissue on the treated side compared to the untreated side after 5 min of treatment. (H&E, Longitudinal section, 2X).

Microscopic evaluations revealed progressive changes in both the peritendinous tissue and fibrous tissue structure of the bovine Soleus component of Achilles tendons subjected to PUTT. In the control group, the peritendinous tissue remained intact, with no signs of separation or damage, serving as a baseline for comparison. After 1 min of treatment, minimal changes were observed in the peritendinous tissue, characterized by slight loosening, while the fibrous structure showed minimal disruption, maintaining overall organization. By the 3-min mark, moderate separation in the peritendinous tissue began to manifest, signaling increased tissue disruption, accompanied by initial signs of fibrous tissue misalignment and spacing irregularities (Fig. 2). This disruption progressed by the 5-min treatment, where further thickening and pronounced separation of the peritendinous tissue were noted, along with noticeable separation and change of tendon fibers. The most substantial changes were evident after 7 min of treatment, where there was marked thickening and extensive separation of the peritendinous tissue, occasionally resulting in the loss of this layer (Table 2). Correspondingly, significant changes in tendon fibrous structure were observed, characterized by distinct fiber disruption compared to the control side of the tissue (Fig. 3). Additionally, an increase in targeted tendon fibrous tissue modification was observed with prolonged PUTT application times.

**Table 2:** Targeted modifications in peritendinous tissue and tendon fibrous structure across treatment durations.

Treatment duration	Peritendinous tissue condition	Peritendinous observations	Tendon fibrous tissue conditions	Tendon fibrous tissue observations
1 Minute	Minimal changes	Slight loosening, no significant separation	Minimal modification	Subtle modifications in tissue organization
3 Minutes	Moderate changes	Moderate separation, indicative of targeted modification	Initial modification	Initial alignment adjustments and spacing modifications
5 Minutes	Pronounced changes	Increased thickening and pronounced separation	Noticeable modification	Increased separation and targeted modified of tendon fibers
7 Minutes	Extensive changes	Marked thickening and extensive separation, occasional loss of the paratenon	Extensive modification	Comprehensive tissue modification with widespread separation of fibrous tissue integrity

### 3.3 Comparative analysis and photographic documentation

**Fig. 4:**
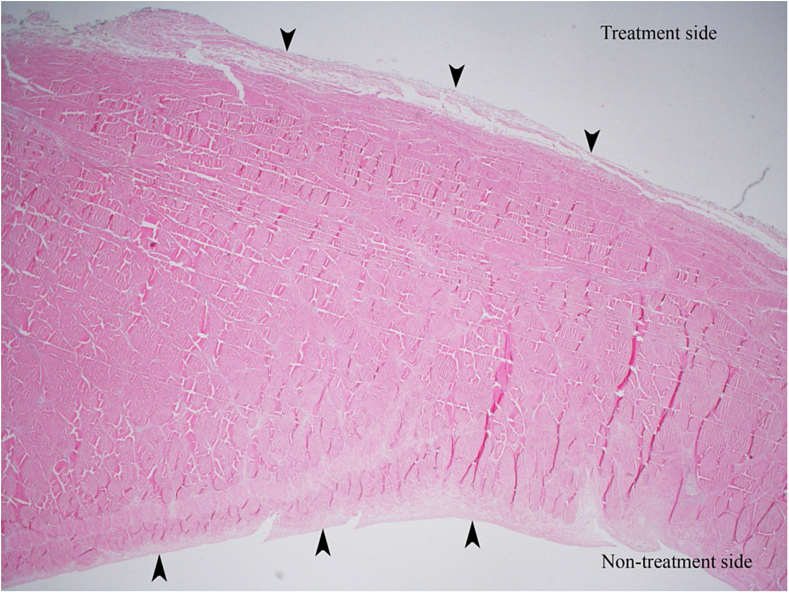
Paratenon Separation Following PUTT — This microscopic image illustrates the structural differences between the PUTT-treated and non-treated sides of a tendon. Black arrows highlight the paratenon layer, delineating notable separation from the tendon's fibrous layer in the treated region. (H&E, Cross Section, 2X).

**Fig. 5:**
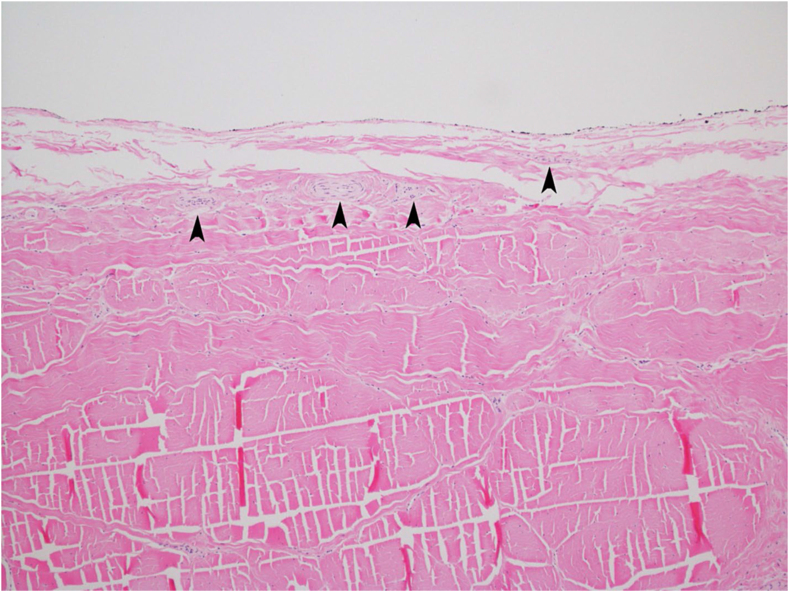
Vascular and Neural Presence in the Paratendinous Layer — This image reveals the presence of small vessels and nerve fibers within the paratendinous layer at the treated area. (H&E, Cross Section, 10X).

A series of macroscopic and microscopic images meticulously documented the changes occurring across various treatment durations. Fig. 4 displays the paratenon structural differences between the PUTT-treated side and the non-treated side of a tendon, emphasizing the extent of paratenon separation following PUTT treatment. Fig. 2 shows progressive levels of paratenon separation, providing detailed macroscopic views that align with microscopic observations to offer a comprehensive understanding of tissue modifications. These figures illustrate a treatment time-dependent increase in the extent of separation, visually capturing the loosening of the paratenon and correlating each stage with the duration of PUTT application. Notably, the images from the 7-min treatment group showcased the most loosening area of paratenon, emphasizing the significant impact of prolonged treatment. Furthermore, microscopic analysis revealed the presence of small vessels and nerve fibers within the paratenon layer (Fig. 5).

### 3.4 Measurement of penetration depth and statistical analysis

This analysis aimed to quantify the degree of tissue modification in the tendon fibrous layer following PUTT. The results demonstrated a progressive increase in penetration depth with longer treatment durations. Specifically, the mean penetration depths recorded were 0.1 mm (SD = 0.01 mm) for the 1-min duration, 2.57 mm (SD = 0.02 mm) for 3 min, 2.61 mm (SD = 0.17 mm) for 5 min, and 3.93 mm (SD = 0.13 mm) for 7 min, indicating an increasing degree in tissue modification (Table 3).

**Table 3:** Summary of penetration depths across treatment durations.

Treatment duration	Mean of penetration depth (mm)	Standard deviation (mm)
1 Minute	0.10	0.01
3 Minutes	2.57	0.02
5 Minutes	2.61	0.17
7 Minutes	3.93	0.13

**Fig. 6:**
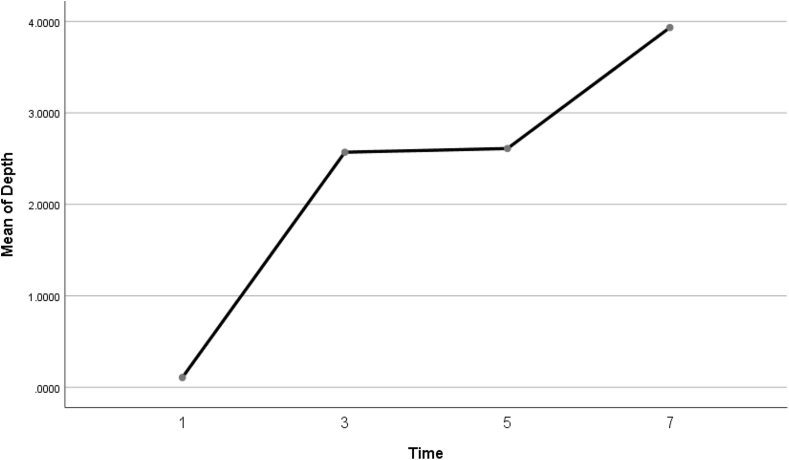
Mean penetration depth by treatment duration — This line graph displays the mean depth of tissue penetration (in millimeters) across four treatment durations (1, 3, 5, and 7 min). It illustrates a progressive increase in penetration depth with extended treatment durations, emphasizing the dose-dependent effect of PUTT on tissue modification.

The difference in penetration depths across treatment durations (1, 3, 5, and 7 min) was analyzed using an Analysis of Variance (ANOVA), which showed significant differences among groups (F(3, 8) = 620.898, p < 0.001). The eta squared (η^2^) was 0.996, indicating a large effect size. This confirms the strong influence of treatment duration on tissue modification. Post hoc comparisons using Tukey's Honest Significant Difference (HSD) test highlighted significant differences between most groups (p < 0.001), except between the 3-min and 5-min treatments, where no significant difference was observed (p = 0.969). These findings support a dose-dependent relationship between treatment duration and tendon structural modifications, with increasing penetration depths correlating with longer treatment times. Fig. 6 illustrates these findings, displaying a graphical representation of the progressive increase in mean penetration depths across the treatment durations, highlighting the dose-dependent impact of PUTT.

## 4 Discussion

The results of this study demonstrate that Percutaneous Ultrasound Tenotomy (PUTT) induces significant time-related structural changes in both the tendon's fibrous layer and surrounding paratenon. Our findings reveal a clear increase in tendon penetration depth and paratenon separation with longer treatment durations, as confirmed by both macroscopic and microscopic analyses. These findings demonstrate that the extent of tissue modification is directly related to the duration of PUTT, suggesting a dose-dependent structural impact. This relationship highlights the progressive influence of longer treatment durations on tissue integrity and consistency. The structural changes reported in the current study provide histological evidence supporting the mechanism of action of PUTT. The findings in the current study corroborates with previous studies regarding the mechanism of action of PUTT through which it may disrupts pathological nerve ingrowth and structural changes to tendon architecture [20,23,24]. This potential mechanism, though not previously reported as a mechanism, may support the efficacy of PUTT in improving chronic tendon pain, as observed in several clinical studies and systematic reviews [7,[11], [12], [13],^25^]. By mechanically separating the paratenon, PUTT could disrupt nociceptive fibers, which is known to occur with other percutaneous procedures [21,[23], [24], [25], [26]]. This process may also affect vascular and neural components of the tendon embedded within the paratenon, potentially contributing to neuromodulatory effects and pain modulation [20,[27], [28], [29], [30]]. Similarly, surgical techniques such as open revision, endoscopic debridement, and minimally invasive stripping are based on comparable hypotheses but may not be suitable for patients who are not surgical candidates [25,26,[30], [31], [32]]. In this context, PUTT stands as a promising option for patients unresponsive to conservative management and ineligible for surgery.

Although PUTT (TENEX®) has received FDA approval for chronic tendinopathy and is supported by clinical evidence, establishing a standardized protocol that ensures both efficacy and safety remains essential. Our study highlights the potential for shorter treatment durations to achieve effective anti-nociceptive outcomes, but a longer duration of PUTT may be necessary for modifying tendon elasticity. This may be due to a biological threshold where structural changes induced by ultrasonic energy reach a plateau [33]. Notably, although a progressive increase in tendon penetration depth was observed across treatment durations, the difference between the 3-min and 5-min groups was not statistically significant, possibly indicating a short-term biological threshold. However, a significant increase was observed between the 5-min and 7-min groups, suggesting that prolonged application continues to enhance tissue disruption. Clinically, this may support a tiered approach, shorter durations for paratenon separation and pain modulation, and longer durations when deeper structural modification is needed. Future biomechanical studies with larger sample sizes will be important for confirming these findings and refining treatment protocols to further optimize PUTT's efficacy. Another area of important investigation will be to better understand the effect of PUTT's ultrasound energy on nociceptor physiology, as ultrasound is already an accepted model for treating various pathologies [[34], [35], [36]].

Our results suggest that a 3-min application may be adequate for alleviating chronic tendon pain through paratenon separation, a fibrous tissue that is densely populated with Substance P and CGRP peptidergic nerves, which are known to be chronic pain modulators [16,28,37]. However, our results also suggest that PUTT may be able to perform a partial tendon release, as we achieved nearly 4 mm of tendon parenchyma penetration and disruption at 7 min of working time. This may be important when considering the performance of a release in some patients with stiff or shortened tendons. Personalized protocols should consider patient pain intensity, tendon thickness integrity, and length to maximize therapeutic benefits while minimizing the risk of tendon rupture. This tailored approach is critical for optimizing treatment protocols across various indications, as it can reduce discomfort and risks associated with prolonged treatment without compromising efficacy.

Future research should aim to refine treatment protocols by investigating the optimal duration and intensity of PUTT based on tendon pain, Comparative studies evaluating different PUTT protocols will further help expand its clinical indications and fine-tune these strategies for broader application. In vivo, studies exploring the neuromodulatory changes within the paratenon induced by short-term PUTT could provide valuable insights into the underlying neural physiology of chronic tendon pain. Additionally, further studies are needed to explore the elastic and tensile properties of tendons with longer-term PUTT (>7min) to opine in its clinical applicability for tendon lengthening.

One notable limitation of this study is the removal of the tendon from its native bone insertion prior to treatment. While this approach allowed for standardized treatment application and consistent histological sampling across specimens, it does not fully replicate the in situ physiological environment of clinical PUTT procedures. The fixed configuration helped ensure reproducibility of treatment depth, angle, and exposure, but future studies should consider an intact model that better mirrors clinical conditions, preserving tendon-bone continuity to enhance translational value.

## 5 Conclusion

This study provides important insights into the possible mechanisms underlying the results of PUTT in a myriad of clinical studies. By clarifying the dose-dependent effects and determining optimal treatment durations, this research aids in the development of safer and more effective patient-specific therapeutic strategies. Ongoing investigations into the mechanisms and outcomes of PUTT with larger sample sizes will be essential for its successful integration into clinical practice, providing promising solutions for patients with chronic tendon disorders. However, the small sample size used in this study may have limited the statistical power to detect subtle differences between treatment durations. Nonetheless, these findings establish a crucial foundation for further exploration of PUTT.

## Data availability

Data sharing not applicable to this article - no new data generated or the article describes entirely theoretical research.

## Funding sources

This research did not receive any specific grant from funding agencies in the public, commercial, or not-for-profit sectors.

## Declaration of competing interest

Authors provided Conflict of Interest forms for all authors.
